# Evaluation of Attitudinal Beliefs Held by Medical and Nursing Students Towards Suicidal Behavior

**DOI:** 10.3390/nursrep14040261

**Published:** 2024-11-18

**Authors:** Thelma Beatriz González-Castro, María Lilia López-Narváez, Ana Fresán, Carlos Alfonso Tovilla-Zárate, Alma Delia Genis-Mendoza, Humberto Nicolini, Juan Pablo Sánchez de la Cruz, Yazmín Hernández-Díaz

**Affiliations:** 1 División Académica Multidisciplinaria de Jalpa de Méndez, Universidad Juárez Autónoma de Tabasco, Jalpa de Méndez, Tabasco 86025, Mexico; thelma.glez.castro@gmail.com (T.B.G.-C.); yazmin.hdez.diaz@gmail.com (Y.H.-D.); 2 División Académica Multidisciplinaria de Comalcalco, Universidad Juárez Autónoma de Tabasco, Comalcalco, Tabasco 86025, Mexico; dralilialonar@yahoo.com.mx; 3 Subdirección de Investigaciones Clínicas, Instituto Nacional de Psiquiatría Ramón de la Fuente Muñiz, Ciudad de México 14370, Mexico; a_fresan@yahoo.com.mx; 4 Servicio de Atención Psiquiátrica, Hospital Psiquiátrico Infantil Dr. Juan n. Navarro, Ciudad de México 14080, Mexico; 5 Laboratorio de Genómica de Enfermedades Psiquiátricas y Neurodegenerativas, Instituto Nacional de Medicina Genómica, Ciudad de México 14610, Mexico; hnicolini@inmegen.gob.mx; 6 Salud Ocupacional e Higiene, Planta de Concretos Cancún 1, Holcim México Operaciones, Cancún 05348, Mexico; juanpablo.sanchezdelacruz@holcim.com

**Keywords:** nursing, medical, students, suicide, attitudinal beliefs

## Abstract

Background/Objectives: A favorable attitude towards suicidal behavior is associated with an increased risk of suicidal behavior in youth populations. Hence, the aim of the present study was to analyze attitudinal beliefs about suicidal behavior among Mexican medical and nursing students. We also compared attitudinal beliefs about suicidal behavior according to the religious affiliation of the participants. Methods: This is a cross-sectional observational study. Attitudinal beliefs about suicidal behavior were assessed using the Attitudinal Beliefs Questionnaire about Suicide Behavior (CCCS-18). We evaluated personal and family histories of suicide using the Spanish version of the MINI International Neuropsychiatric Interview. Comparative analysis between nursing and medical students was performed, using Chi-square tests for categorical variables and Student *t*-tests for continuous variables. Results: A total of 195 (52.2%) medical students and 178 (47.8%) nursing students participated. Medicine students reported a higher prevalence of a family history of suicide attempts and knowing someone who had tried to die by suicide compared to nursing students (*p* = 0.001). Regarding attitudinal beliefs—specifically, suicide in terminal patients—medical students reported higher scores than nursing students (9.50 ± 5.91 vs. 11.23 ± 6.38, *p* < 0.001), while the latter exhibited higher scores in attitudinal beliefs related to suicide itself (9.55 ± 4.45 vs. 7.28 ± 4.09, *p* < 0.001). Both groups display similar scores when compared by religious affiliation. Conclusions: Our findings show differences in attitudinal beliefs about suicidal behavior between medical and nursing students. Medical students exhibited more positive responses toward suicide in terminal patients, while nursing students had higher values for attitudinal beliefs related to suicide itself. These results could be considered in the planning of health sciences curricula in order to positively impact future suicide prevention efforts. This study was retrospectively registered at the Universidad Juarez Autónoma de Tabasco, with the registration number 20240063 on 8 June 2024.

## 1. Introduction

Every year, nearly 800,000 people die by suicide. The incidence of suicide deaths is higher in developing countries than in developed countries [1]. The magnitude and seriousness of suicidal behavior continue to make suicide a leading cause of death worldwide. For example, epidemiological data show a mortality rate of 11.3 per 100,000 in Poland and a rate between 10.3 and 16.3 per 100,000 in India, with a higher risk in men than in women [2,3]. Furthermore, North, Central, and South America have experienced a significant increase in suicide mortality over the past 20 years [4]. Furthermore, death by suicide is the second cause of death among individuals aged 10–14 and 25–34 years in the United State, and the third cause of death among individuals aged 15–24 years [5,6]. In Mexico, death by suicide has increased over the last 5 years, particularly among young populations aged 15–24 years [7]. Also, recent studies reported a 600% increase in suicide attempts among Mexican adolescents from 2006 to 2022 [8].

Despite its importance in terms of public health, suicidal behavior is often met with misunderstanding and negativity. It has been suggested that actions to prevent deaths by suicide should include training healthcare personnel to detect and treat the risk of suicide in patients [9,10]. Healthcare professionals play a pivotal role in the support and prevention of mental illnesses, including suicide [11]. This is especially important as healthcare providers influence the motivation to treat patients during a suicidal crisis [11,12]. However, attitudes towards suicidal behavior may differ between health professions, such as nurses and doctors [12,13]. The transition from adolescence to adulthood is a significant period of psychological change, during which suicidal ideation is a critical issue to address in medical and nursing students, who face numerous stressors during their education. Health students need to acquire skills to manage the stressors they may encounter during their academic formation, as these can affect their mental health and well-being [14,15].

Moreover, it has been suggested that stressful events are associated with an increased risk of suicidal behavior, while spiritual experiences may provide protection against suicidal behavior [16,17]. Spirituality has been observed as a protective factor in university students in several regions of the world [18,19,20]. Nevertheless, the role of spiritually as a protective factor against suicidal behavior has not been widely studied. Medical and nursing students are often exposed to several factors that could increase their stress. Studies show that individuals may respond to suicidal behavior based on their personal attitudes toward it [21]. Therefore, it is possible that a lack of knowledge and negative attitudes toward suicide can have a negative impact on healthcare care and individual’s safety [22].

Negative attitudes toward suicidal behavior are associated with a lack of professional preparation, stigma, and even discrimination, which may impair the quality of patient care. Therefore, it is essential to examine attitudinal beliefs about suicide in healthcare students [23]. This would enable the development of strategies to improve their academic experience and, consequently, the quality of care and patient safety provided when they begin clinical practice and social service. Similarly, attitudes toward suicide are associated with the likelihood of a suicide attempt or death by suicide.

Suicidal behavior is more prevalent among healthcare workers than in the general population [24]. Depression, anxiety, and suicidal behavior have also been observed in nursing students [25]. Given these findings, it is important to evaluate the attitudes of nursing and medical students toward suicide prevention [26]. However, many factors remain unclear, such as the role of attitudinal beliefs, which are expressed in attitudes toward suicide. The CCCS-18 (Attitudinal Beliefs Questionnaire about Suicide Behavior) is an effective tool with which to evaluate and detect self-critical thoughts, as well as to assess their progression before an attempt is made [27]. Therefore, the aim of the present study is to measure the attitudinal beliefs about suicide behavior in nursing and medical students. Furthermore, we consider it important to assess whether the religiosity of healthcare students has an impact on their attitudinal beliefs about suicidal behavior.

## 2. Materials and Methods

### 2.1. Ethics Statement

First, this study was submitted for consideration to the ethics committee of the Juarez Autonomous University of Tabasco, which approved the study. Second, all students included in the study received detailed information about the objectives of the study. Students who decided to participate read and signed an informed consent form. Participation in the study was voluntary and participants did not receive any financial remuneration.

### 2.2. Design of This Study

This is a school-based cross-sectional conducted among students from public schools in Comalcalco, Tabasco, México. The present study used a non-probabilistic sampling approach.

### 2.3. Participants

In the present study, students from all academic levels were included to ensure a balanced representation of information. We assessed students from a faculty of the University of Tabasco, Mexico.

### 2.4. Assessment Procedure

Data collection took place from March to July 2024, during the spring semester. A semi-structured data sheet was designed. The first section collected and assessed sociodemographic information, personal characteristics, lifestyle habits, and other factors, which were documented. Sociodemographic variables included sex, marital status, socioeconomic status, and participation in other activities. The attendance of religious services, including the specific religion followed by the student, was also inquired. This question was based on the cultural context and the most commonly practiced religions in the region (INEGI) [28].

The students were asked about their personal and family history of suicide. To assess personal suicide history, we used the MINI International Neuropsychiatric Interview in Spanish, as reported elsewhere [26]. For the family history of suicide attempts, we included questions such as “Do you have a sibling who has attempted to die by suicide?” and “Do you have a parent who has attempted to die by suicide?” For the broader family history of suicide, we asked “Do you know anyone who has tried to die by suicide?” and “Do you know anyone who has died by suicide?” The questionnaire was administered face-to-face by trained interviewers.

### 2.5. Attitudes Toward Suicidal Behavior

Attitudes toward suicidal behavior were assessed using the Attitudinal Beliefs Questionnaire about Suicide Behavior (CCCS-18) [27]. This instrument was validated in Spanish [29]. The CCCS-18 comprises 18 items rated on a 7-point Likert scale (ranging from 1 = strongly disagree to 7 = strongly agree) and has 4 factors: the legitimization of suicide (Cronbach’s α = 0.84), suicide in terminal patients (Cronbach’s α = 0.82), the moral dimension of suicide (Cronbach’s α = 0.78), and suicide itself (Cronbach’s α = 0.73). The legitimization of suicide refers to the acceptance of suicidal behavior, with higher scores reflecting greater acceptance. Death by suicide in terminal patients reflects the acceptance of suicide in individuals with no further possibilities for survival. Regarding the moral dimension of suicide, higher scores indicate disagreement with moral statements. Finally, higher scores on suicide itself reflect the view that suicide is an escape from a given situation [30].

### 2.6. Statistical Analysis

Demographics, religious service attendance, and suicide history are presented, with frequencies and percentages given for categorical variables. All continuous variables are presented with means and standard deviation (SD) values. The Kolmogorov–Smirnov test was used to analyze whether continuous variables followed a normal distribution, with a *p*-value of *p* > 0.08 indicating normality. The comparison of categorical variables between nursing and medical students was performed with Chi-square tests, while Student *t*-tests were used for the comparison of continuous variables [31]. A multivariate general linear model was used to identify differences in attitudinal beliefs about suicidal behavior between nursing and medical students and by religion.

To determine the power of the analysis, we used the G*Power software, Version 3.1.9.7 (Heinrich Heine Universität Düsseldorf, Düsseldorf, Germany) specifying two-tailed tests and an effect size (d) of 0.5. The sample size for medical students was 195 participants and that for nursing students was 178, with a power of 0.98. The significance for all tests was set to *p* ≤ 0.05.

## 3. Results

### 3.1. Description of the Sociodemographic and Academic Features

This study included 373 students. Of these, 195 (52.2%) were medical students and 178 (47.8%) were nursing students. The majority of the subjects in our sample were women (n = 229, 61.4%) and single (n = 351, 64.1%), which reflects the fact that most nursing students are women. The majority of the sample were university students who also engaged in activities outside of their academic life (n = 320, 85.8%). These activities included full-time employment (n = 159, 42.6%), part-time employment (n = 119, 31.9%), participation in sports groups (n = 31, 8.4%), and involvement in active religious roles (n = 11, 2.9%). Most of the sample (n = 334, 90.3%) attended religious services. The majority identified as Catholic (n = 214, 57.5%), followed by Pentecostal and Presbyterian (n = 92, 24.7%), Adventist, or Jehovah’s Witness (n = 30, 8.1%). Only 36 students (9.7%) did not report any religious affiliation (See Table 1).

### 3.2. Personal History of Suicide Behavior Details

A description of the personal history of suicidal behavior in nursing and medical students is shown in Table 2. Medical students (n = 33, 16.9%) had a higher family history of suicide attempts compared to nursing students (n = 15, 8.4%). When asked whether they knew anyone who had tried to die by suicide, medical students (n = 98, 50.3%) were more likely to respond affirmatively than nursing students (n = 59, 33.1%).

### 3.3. Attitudinal Beliefs on Suicide Behavior by Groups

The comparative analysis of attitudinal beliefs about suicidal behavior between nursing and medical students revealed differences in the attitudinal beliefs of the two groups. The medical student group showed higher values for “suicide in terminal patients”, while the nursing group had higher values for “suicide itself”. No differences between groups were observed in terms of the “legitimization of suicide” and the “moral dimension of suicide” (see Table 3).

### 3.4. Attitudinal Beliefs on Suicide Behavior by Religion of the Participant

In the comparison of groups based on the participant’s religion, similar scores were found between groups (see Table 4).

Finally, we assessed whether the attitudinal beliefs could be modified by the interaction between a nursing or medical student and the participant’s religion. No interaction effects were found (see Table 5).

## 4. Discussion

The objectives of the present study were to evaluate the attitudinal beliefs about suicidal behavior among medical and nursing students. Additionally, we analyzed these beliefs according to participants’ religion. Healthcare workers play a pivotal role in suicide prevention; however, it has been shown that sometimes they hold unfavorable attitudes toward suicide. Therefore, it is crucial to understand attitudes and beliefs about suicide in medical and health science students [32], as detecting unfavorable attitudes toward suicide could help to develop strategies to improve their educational formation. Thus, the findings presented in this study are highly relevant.

When recording the personal history of suicide, medical students reported a higher percentage of positive responses regarding a family history of suicide attempts and knowing someone who had tried to die by suicide. This personal background is important to highlight as it has a direct link to attitudinal beliefs. For example, it has been reported that individuals who had experienced a suicide within their immediate family or among other relative are more likely to believe that suicide is unpreventable than those who have not had this experience [33,34]. Based on this, we could hypothesize that such individuals may become desensitized to the emotionally painful event, which could reduce their fear of death. Therefore, it is important to consider the family history of suicide and exposure to suicide attempts when designing professional education programs for mental health [15].

Our findings recognize a primary difference between nursing and medical students. Nurses are more likely to accept the idea of suicide, while medical students show more positive attitudes toward suicide in terminal patients. This highlights the importance of incorporating specific curricula tailored to each profession to improve mental health education, which will directly impact future patient care [35,36]. Furthermore, it is well documented that gender can be a significant predictor of psychological health. Epidemiological data show that women experience suicidal behavior at higher rates than men [37,38]. Given that nursing is predominantly performed by women, it would be beneficial to include topics in the nursing curriculum that address these gender-related differences [39].

Additionally, our results show that medical students have a more positive attitude toward suicide in terminal patients than nursing students, aligning with findings reported in the literature. For example, medical students from Servia were found to have a more favorable attitude toward euthanasia [40]. This suggests that proper education in palliative care and end-of-life topics could enhance the communication skills of medical students.

On the other hand, a particularly interesting finding is that nursing students had a more positive attitude toward suicide itself than medical students. It is necessary to consider that nursing students may face pressure from colleagues, supervisors, and teachers, which can increase stress and potentially lead to depression [41]. Thus, it is essential to assess both beliefs and attitudes toward suicide in order to guide prevention programs and curricula for medical and nursing students, especially for those nursing students who exhibit high levels of positive attitudes toward suicide itself. This could help to prevent deaths by suicide [42]. Attitudes toward suicide are very complex, involving multiple factors such as genetics, biology, social influences, and the environment. In our study, we found that nursing students (73%) were predominantly women compared to medical students (50.8%). There is evidence supporting marked differences between men and women regarding attitudes toward suicide prevention, with men generally having more positive attitudes toward preventative measures [43]. Consequently, it is proposed that the attitudes of nursing and medical students toward suicide influence their motivation to treat patients in suicidal crises [22].

There is growing evidence that religiosity directly impacts mental health outcomes [16,44,45]. In this study, we evaluated the relationship between religion and attitudinal beliefs about suicide. Despite Mexico’s high religious population and the powerful influence of religion on the nation’s culture [46], we did not find a correlation between religiosity and attitudes toward suicide. One explanation for this could be the homogeneity of religious practice in Mexico, where most religious affiliations are Christian. Religious beliefs may be deeply embedded in everyday life, making it difficult to detect a distinct statistical association with attitudes toward suicide. Previous studies suggest that religiosity is linked to suicide prevention, as it provides a support system that can reduce the likelihood of suicide behavior [18,47,48]. However, in the present study, no significant differences were found between the religious affiliations of nursing and medical students. This could be due to the cultural normalization of religion in Mexico, where religious practices are common across many sectors of the society.

We would like to highlight some limitations of the study. Our sample was limited to students from the Juarez Autonomous University of Tabasco, a public university in Mexico. Therefore, the findings are not representative of all students enrolled in universities across Mexico. Furthermore, since the data were self-reported, there may be potential biases due to social desirability, leading to inaccuracies arising from underreported information. Moreover, as a cross-sectional study, we can only establish associations and cannot infer causality. Nevertheless, the findings provide valuable insights into attitudinal beliefs about suicide. Future research employing different methodologies is needed to further explore the challenges faced by nursing and medical students in Mexico.

## 5. Conclusions

To sum up, medical students showed a more positive response to suicide in terminal patients compared to nursing students. On the other hand, nursing students had a more positive response toward suicide itself. These differences should be considered when planning the curriculum for health science students. Additionally, factors such as gender or religious practice should be taken into account when evaluating attitudinal beliefs about suicidal behavior. Addressing these differences can help to improve suicide prevention efforts and future patient care.

## Figures and Tables

**Table 1:** Sociodemographic and academic characteristics of the student participants.

Characteristics	All Sample	Nursing n = 178	Medicine n = 195	Statistics
Sex				x2 = 19.45, *p* < 0.001
Men	144 (38.6%)	48 (27.0%)	96 (49.2%)	
Women	229 (61.4%)	130 (73.0%)	99 (50.8%)	
Marital Status				x2 = 2.62, *p* = 0.26
Single	351 (94.1%)	164 (92.1%)	187 (53.3%)	
Married/living with a partner	18 (4.8%)	11 (6.2%)	7 (3.6%)	
Divorced/separated	4 (1.1%)	3 (1.7%)	1 (0.5%)	
Additional activities to study				x2 = 47.82, *p* < 0.001
Yes	320 (85.8%)	176 (98.9%)	144 (73.8%)	
No	53 (14.2%)	2 (1.1%)	51 (26.2%)	
Type of activity				x2 = 61.06, *p* < 0.001
Full-time employment	159 (42.6%)	120 (67.4%)	39 (20.0%)	
Part-time employment	119 (31.9%)	49 (27.5%)	70 (35.9%)	
Sports	31 (8.4%)	5 (2.8%)	26 (13.3%)	
Religious	11 (2.9%)	2 (1.1%)	9 (4.6%)	
Socioeconomic level				x2 = 16.7, *p* < 0.001
High	12 (3.2%)	6 (3.4%)	6 (3.1%)	
Medium	294 (78.8%)	125 (70.2%)	169 (86.7%)	
Low	67 (18.0%)	47 (26.4%)	20 (10.3%)	
Have you failed any school subject?				
Yes	159 (42.6%)	66 (37.1%)	93 (47.7%)	x2 = 4.28, *p* = 0.03
No	214 (57.4%)	112 (62.9%)	102 (52.3%)	
Attendance of religious services				
Yes	336 (90.3%)	153 (85.9%)	183 (93.7%)	x2 = 6.48, *p* = 0.01
No	37 (9.7%)	25 (14.1%)	12 (6.2%)	
Religion identified with				
Pentecostal and Presbyterian	92 (24.7%)	33 (18.6%)	59 (30.3%)	x2 = 11.03, *p* = 0.01
Catholic	214 (57.5%)	107 (60.5%)	107 (54.8%)	
Adventist or Witness	30 (8.1%)	13 (7.3%)	17 (8.7%)	
Not religion	36 (9.7%)	24 (13.4%)	12 (6.1%)	
Age	20.47± 1.84	19.80 ± 1.53	21.08 ± 1.89	t = −7.21, *p* < 0.001

**Table 2:** Personal history of suicidal behavior.

	All Sample	Nursing n = 178	Medicine n = 195	Statistics
Personal history of suicide attempt	42 (11.3%)	20 (11.2%)	22 (11.3%)	x2 = 0, *p* = 1
Family history of suicide attempt	48 (12.9%)	15 (8.4%)	33 (16.9%)	x2 = 5.99, *p* = 0.01
Family history of death by suicide	22 (5.9%)	11 (6.2%)	11 (5.6%)	x2 = 0.04, *p* = 0.82
Do you know anyone who tried to die by suicide?	157 (42.1%)	59 (33.1%)	98 (50.3%)	x2 = 11.17, *p* = 0.001
Do you know anyone who died by suicide?	138 (37.0%)	64 (36.0%)	74 (37.9%)	x2 = 0.15, *p* = 0.69

**Table 3:** Comparative analysis of attitudinal beliefs on Suicidal Behavior between nursing and medical students.

Attitudinal Beliefs	All Sample Mean ± SD	Nursing n = 178 Mean ± SD	Medicine = 195 Mean ± SD	Statistic
Legitimization of suicide	12.18 ± 6.11	12.48 ± 5.98	11.98 ± 6.23	t = 0.90, *p* = 0.36
Suicide in terminal patients	10.44 ± 6.22	9.50 ± 5.91	11.23 ± 6.38	t = −2.80, *p* = 0.005
Moral dimension of suicide	12.98 ± 4.35	12.97 ±4.50	13.00 ± 4.22	t = −0.05, *p* = 0.96
Suicide itself	8.36 ± 4.41	9.55 ± 4.45	7.28 ± 4.09	t = 5.12, *p* < 0.001

**Table 4:** Comparison of attitudinal belief on suicide behavior by religion.

Attitudinal Beliefs	Pentecostal and Presbyterian Mean ± SD	Catholic Mean ± SD	Adventist or Witness Mean ± SD	Non-Religious Mean ± SD	Statistic	
					F	*p*-Value
Legitimization of suicide	12.84 ± 6.90	12.08 ± 5.87	11.90 ± 6.88	11.41 ± 4.74	0.54	0.65
Suicide in terminal patients	10.87 ± 6.60	10.12 ± 6.05	11.56 ±6.55	10.33 ± 6.06	0.65	0.58
Moral dimension of suicide	13.07 ± 4.58	12.99 ± 4.20	13.30 ± 4.54	12.75 ± 4.37	0.09	0.96
Suicide itself	8.15 ± 4.56	8.56 ± 4.49	8.10 ± 4.29	8.08 ± 3.69	0.29	0.83

**Table 5:** Variable effects using multivariate ANOVA.

Factor/Dependent Variable	F	*p*-Value	Power
Nursing or medical student			
Legitimization of suicide	0.64	0.42	0.12
Suicide in terminal patients	6.15	**0.01**	0.69
Moral dimension of suicide	0.99	0.32	0.16
Suicide itself	14.64	**0.001**	0.96
Religion of the participant			
Legitimization of suicide	0.84	0.47	0.23
Suicide in terminal patients	0.32	0.80	0.11
Moral dimension of suicide	0.06	0.97	0.06
Suicide itself	0.34	0.79	0.11
Nursing or medic student x religion of the participant			
Legitimization of suicide	0.32	0.81	0.11
Suicide in terminal patients	2.48	0.06	0.61
Moral dimension of suicide	1.59	0.19	0.41
Suicide itself	0.06	0.98	0.06

Bold type denotes significant values.

## Data Availability

The data are available under request.

## Public Involvement Statement

No public involvement in any aspect of this research.

## Guidelines and Standards Statement

This manuscript was drafted against the STROBE Statement.
